# Genome Scans Reveal Homogenization and Local Adaptations in Populations of the Soybean Cyst Nematode

**DOI:** 10.3389/fpls.2018.00987

**Published:** 2018-07-17

**Authors:** Anne-Frédérique Gendron St-Marseille, Etienne Lord, Pierre-Yves Véronneau, Jacques Brodeur, Benjamin Mimee

**Affiliations:** ^1^Saint-Jean-sur-Richelieu Research and Development Centre, Agriculture and Agri-Food Canada, Saint-Jean-sur-Richelieu, QC, Canada; ^2^Institut de Recherche en Biologie Végétale (IRBV), Université de Montréal, Montréal, QC, Canada

**Keywords:** Bayesian outlier detection, genetic diversity, genotyping-by-sequencing, *Heterodera glycines*, isolation by distance

## Abstract

Determining the adaptive potential of alien invasive species in a new environment is a key concern for risk assessment. As climate change is affecting local climatic conditions, widespread modifications in species distribution are expected. Therefore, the genetic mechanisms underlying local adaptations must be understood in order to predict future species distribution. The soybean cyst nematode (SCN), *Heterodera glycines* Ichinohe, is a major pathogen of soybean that was accidentally introduced in most soybean-producing countries. In this study, we explored patterns of genetic exchange between North American populations of SCN and the effect of isolation by geographical distance. Genotyping-by-sequencing was used to sequence and compare 64 SCN populations from the United States and Canada. At large scale, only a weak correlation was found between genetic distance (Wright's fixation index, F_ST_) and geographic distance, but local effects were strong in recently infested states. Our results also showed a high level of genetic differentiation within some populations, allowing them to adapt to new environments and become established in new soybean-producing areas. Bayesian genome scan methods identified 15 loci under selection for climatic or geographic co-variables. Among these loci, two non-synonymous mutations were detected in SMAD-4 (mothers against decapentaplegic homolog 4) and DOP-3 (dopamine receptor 3). High-impact variants linked to these loci by genetic hitchhiking were also highlighted as putatively involved in local adaptation of SCN populations to new environments. Overall, it appears that strong selective pressure by resistant cultivars is causing a large scale homogenization with virulent populations.

## Introduction

The introduction of an organism into a new environment can have unpredictable detrimental consequences, including public health problems, losses in biodiversity and ecosystem services or crop yield losses due to exotic weeds, insects and pathogens, altogether resulting in significant economic impact (Pimentel et al., 2005). Unfortunately, the steady increase in international trade facilitates the movement and introduction of new invasive species (Hulme, 2009). In addition, climate change is altering environmental conditions and could change the species' distribution range or favor their establishment in previously unsuitable habitats (Early and Sax, 2014). It is therefore imperative to carry out specific risk assessment in order to target species to be controlled. Alien invasive species, by definition, did not evolve in the biogeographic habitat in which they are introduced. Consequently, they are often poorly adapted to their new environment. Generally, the most successful invaders will have a high potential for rapid adaptation through phenotypic plasticity or microevolution (Novak, 2007). Understanding the genetic mechanisms of local adaptation is therefore critical to predict future species distribution.

Plant-parasitic nematodes are microscopic worms that reduce global annual food production by 12.3% and cause more than US$157 billion in economic losses worldwide (Hassan et al., 2013). In North America, one of the most damaging species is the soybean cyst nematode (SCN), *Heterodera glycines* Ichinohe. Since its first detection in 1954 in Hanover County, North Carolina, SCN has been reported in almost every soybean-producing county in the United States (Davis and Tylka, 2000), as well as in southwestern Ontario, Canada (Anderson et al., 1988). During the 2000s, SCN colonized several new northern localities in the United States (North Dakota in 2003; Mathew et al., 2015) and Eastern Canada (northeastern Ontario in 2007 and Québec in 2013; Mimee et al., 2014). A few studies have investigated SCN dispersal, which seems to follow a northward and eastward pathway in North America (Tylka and Marett, 2017). This dispersion pattern correlates with the expansion of soybean cultivation following the introduction of new soybean varieties with shorter maturity periods and improved tolerance to drought and cold (Shurtleff and Aoyagi, 2010; Yu, 2011).

It is expected that SCN would survive and multiply throughout the current North American soybean-growing area and complete at least two generations at its northern limit (Gendron St-Marseille, 2013). Climate warming should also favor the establishment of SCN at higher latitudes and increase the number of generations per year in most regions. However, these predictions are based strictly on temperature requirements and do not account for the intrinsic capacity of SCN to adapt to new environmental conditions. Genetic variations within a population reflect its evolutionary potential and result from four evolutionary forces that affect individual fitness: mutation, gene flow, selection, and genetic drift (Eizaguirre and Baltazar-Soares, 2014). For most organisms, including SCN, the relative weights of these forces can differ significantly. Mutations are rare events that should not contribute significantly to SCN adaptations in a short time frame. Gene flow depends on the dispersal ability of an organism, which for SCN is achieved mainly by means of human activities at the short spatial scale (Kristjansson, 2010). In addition, wind and flooding can carry SCN cysts over very long distances, and contribute to its dispersal at the regional and continental scales. Many different selection pressures can shape the genetic structure of SCN populations, but host plant is probably the strongest selection factor. The ability of SCN to reproduce on a given soybean genotype differs greatly depending on its resistance genes and the nematode's virulence profile (HG type) (Colgrove and Niblack, 2008; Niblack et al., 2008). Thus, management decisions by growers (for example, the systematic use of resistant cultivars) can result in a strong selection pressure. Finally, the influence of genetic drift will also depend on pest control strategies, because they contribute to dictate population size, although it was shown for cyst nematodes that genetic diversity can be very high within a single cyst (Green et al., 1970). Each nematode female can mate with several males and lay hundreds of eggs that can survive for at least a decade in the soil (Slack et al., 1972). Thus, even if the diversity appears reduced due to genetic drift under strong selection by resistant cultivars, most alleles probably persist for several years in the population in a “dormant” state.

In other cyst nematode species, genetic diversity at the population level has been studied by means of several techniques, including microsatellite markers, ITS-RFLP (internal transcribed spacer–restriction fragment length polymorphism), RAPD (random amplified polymorphic DNA), and 2-DGE (two-dimensional gel electrophoresis) (e.g., Blok et al., 1997; Grenier et al., 2001; Plantard et al., 2008; Boucher et al., 2013). However, these methods focus only on specific sections of the genome, yield very few markers, or do not allow precise comparisons among populations. For example, Eves-van den Akker et al. (2015) recently published a metagenetic approach to genotype populations of the pale cyst nematode, *Globodera pallida*. While this method was shown powerful to rapidly evaluate genetic diversity and distribution of specific mitotypes, it is based on very few neutral markers, which prevent any assessment for selection. Genome scan is an interesting approach for the identification of genetic loci involved in adaptation to specific selection pressure. It was notably used in *G. pallida* to identify genomic regions associated with virulence on resistant potato cultivars (Eoche-Bosy et al., 2017).

Despite rapid advances in next-generation sequencing (NGS) technologies, sequencing a large number of individual genomes at high coverage in order to perform population genetic studies remains very expensive and may require important quantities of DNA from individuals. Elshire et al. (2011) developed a genotyping-by-sequencing (GBS) protocol to rapidly identify single-nucleotide polymorphisms (SNPs). The GBS technique and other restriction-site-associated DNA (RAD) sequencing methods produce large quantities of reads that do not cover the entire genome but have higher sequencing depth, thus reducing sequencing errors (Gautier et al., 2013; Anand et al., 2016). Loci generated by GBS can be present in both coding and non-coding regions and will be shared between all populations owing to the conservation of restriction sites (Cariou et al., 2013). Finally, GBS does not require any prior genomic information for the species being studied, which is an important consideration for SCN since there is no reference genome yet. The optimal gene coverage to reduce the amount of missing data depends on the choice of restriction enzyme (Fu et al., 2016). Fortunately, optimal gene coverage was already tested for the closely related species *Globodera rostochiensis* (Mimee et al., 2015).

The main objectives of this study were to (1) investigate the genetic relationships among SCN populations from United States and Canada, (2) detect isolation by geographical distance (IBD) between SCN populations from United States and Canada, (3) detect genetic loci under selection associated to environmental and climatic parameters, and (4) identify the putative gene functions contributing to the adaptation of SCN populations to specific environmental conditions.

## Materials and methods

### Soybean cyst nematode populations sampling and genotyping-by-sequencing

**Figure 1:**
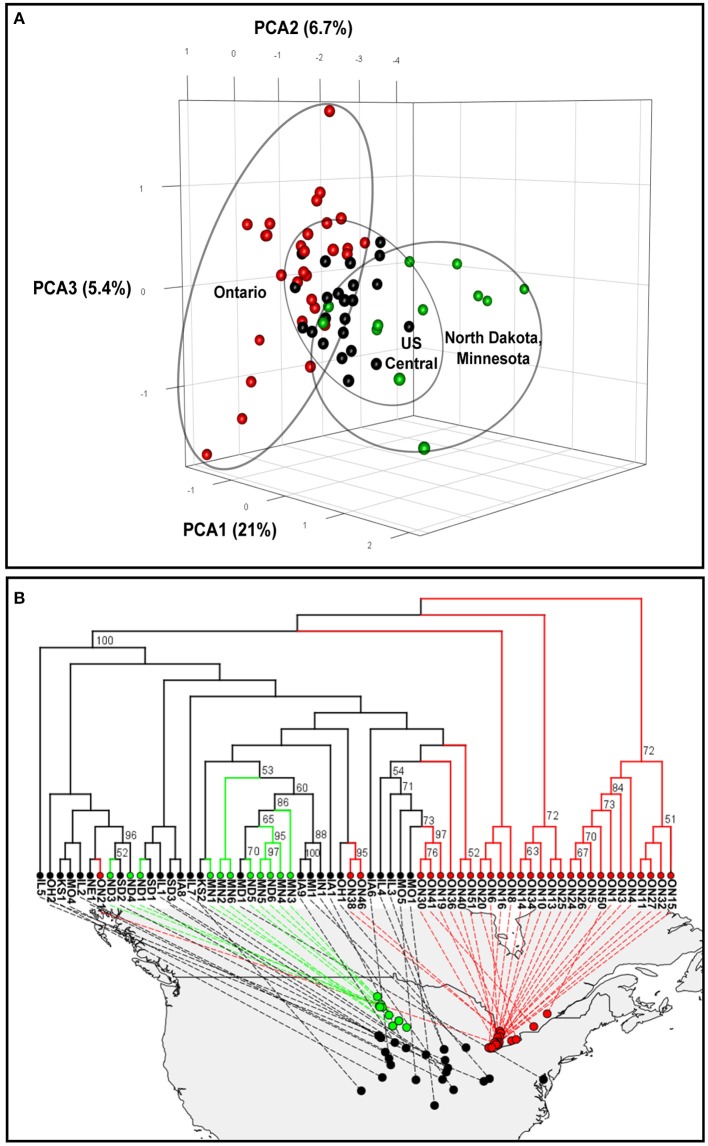
Genetic relationship among North American populations of the soybean cyst nematode. **(A)** Three-dimensional principal component analyses (PCAs). **(B)** Phylogenetic tree plot based on Provesti's absolute genetic distance. Significant bootstrap values (> 50%) are indicated. Soybean cyst nematode populations from Ontario (Canada), North Dakota and Minnesota, and central US states are indicated in red, green and black, respectively.

A total of 64 field populations of SCN, representative of the area currently infested in North America, were sampled or provided by collaborators from 11 US states (Delaware, Iowa, Illinois, Indiana, Kansas, Michigan, Minnesota, Missouri, North Dakota, Ohio, South Dakota) and one Canadian province (Ontario) (Figure 1). For DNA extraction, 40 cysts were randomly chosen from each population and pooled together. Eggs were extracted from each cyst and then washed twice in sterile filtered water. Total genomic DNA of each pool was extracted using the DNeasy Blood and Tissue Kit (Qiagen, Mississauga, ON, Canada) following the manufacturer's instructions. DNA extracts were quantified using Qubit fluorometric quantification (ThermoFisher Scientific, Burlington, ON, Canada) and normalized at 2 ng/μL prior to library preparation and sequencing. These steps were performed following standard protocols (Elshire et al., 2011; Poland et al., 2012) at the Institute for Integrative and Systems Biology (IBIS; Université Laval, Québec City, QC, Canada). Genotyping-by-sequencing was performed using the method described by Mimee et al. (2015) with a combination of two restriction enzymes (PstI/MspI) (New England Biolabs, Whitby, ON, Canada). After the restriction enzyme treatment, all samples (one composite per field population) were barcoded and multiplexed to obtain a single library for the 64 samples, which was sequenced on three Ion Proton chips (ThermoFisher Scientific) at IBIS.

### Single-nucleotide polymorphism calling

The UNEAK pipeline (Lu et al., 2013), which is part of the TASSEL 3.0 bioinformatics analysis package (Bradbury et al., 2007), was used to process the raw reads, since no complete reference genome is yet available for *H. glycines*. This pipeline is designed to call SNPs *de novo*, without a reference genome with high stringency. Only sequences containing a single putative SNP (1-bp mismatch) per sequencing read were kept. Before analysis, the SNPs were further filtered with a minimum coverage (minCov) of 20 reads, a maximum coverage (maxCov) of 10,000 reads, and a minimum allele frequency (MAF) of 0.01.

### Population genetics

Clustering of SCN populations using PCA was carried out using the *prcomp* function from the *stats* package in R software (R Core Team, 2017). The *poppr* package (v2.4.1) in R (Kamvar et al., 2014) was used to investigate the genetic relationship between populations and to build a phylogenetic tree (Provesti's distance, 10,000 bootstraps, neighbor-joining algorithm counting missing data as equivalent in the distance computation). Visualization of sample coordinates and phylogenetic relationship analyses were carried out using the *phytools* 0.6–20 package in R (Revell, 2017).

Fixation index (F_ST_) values (Wright, 1943) were calculated using the classical approach (Hartl and Clark, 2007) with the PoPoolation2 software (Kofler et al., 2011) to evaluate the genetic differentiation between each pair of populations. Furthermore, the effect of isolation by distance on population structure was tested using the correlation between the genetic distance ratio [F_ST_/(1–F_ST_)], as defined by Rousset (1997), and the geographic distance of population pairs in kilometers (km). The geographic distance between each sample location was calculated with the haversine formula using the geosphere package in R. To examine the significance of the relationship between the genetic distance ratio and the pairwise geographic distance distances, we performed a Mantel test (Spearman rank correlation) using the vegan package in R with 999 permutations. To evaluate the effect of time on population genetic differentiation, linear regression analyses were run in R software using pairwise F_ST_ distances based on a point of origin selected on the basis of the first reports of SCN in North America. In our dataset, the closest sample to the oldest population was located in Clarkton, Missouri (MO1) (Hegge, 1957; Tylka and Marett, 2017). To access the genetic isolation from the MO1 location, a Spearman rank correlation (*r*_*s*_) test was performed at a 0.95 confidence level.

Populations from three states (North Dakota, Minnesota, Illinois) were selected as case studies to evaluate the local (short-scale) genetic differentiation. These states were chosen because there were sufficient samples for comparison and because (i) North Dakota shows a recent introduction of SCN and a continuous northward dispersal of the nematode (Nelson and Bradley, 2003; Mathew et al., 2015); (ii) Minnesota has a longer history of SCN, with the nematode being first detected in 1978 and the infested area still expanding each year (Zheng et al., 2006); (iii) in Illinois SCN has been well established in every county for many years (Riggs, 2004; Tylka and Marett, 2017).

### Outlier detection and their association with environmental variables

Two geographic and four climatic covariables were investigated as possible factors explaining loci under selection: latitude (LAT), longitude (LONG), annual mean air temperature (BIO1), maximum air temperature of the warmest month (BIO5), annual precipitation (BIO12), and total precipitation of the warmest quarter (BIO18). All climatic variables were retrieved from the WorldClim global climate database, version 1.4 (Fick and Hijmans, 2017), corresponding to historic conditions (1960–1990). The spatial resolution used for the bioclimatic analysis was set at 30 s or 0.86 km^2^.

To detect correlations between variations in population allele frequencies of SNPs and environmental factors, we used three different Bayesian methods (software programs): BayPass, version 2.1 (Gautier, 2015), BayeScan, version 2.1 (Foll and Gaggiotti, 2008), and BayeScEnv, version 1.1 (de Villemereuil and Gaggiotti, 2015). For each program, triplicate runs with different random seeds were performed with a pilot run of 10,000 iterations to estimate starting parameters, a burn-in length of 50,000 iterations, and a minimum of 50,000 iterations, accounting for the small number of SNPs being investigated. Post-run diagnostics were carried out using the *coda* package in R software in order to ensure sufficient iterations and normality of the Markov chain (Plummer et al., 2006).

The first software, BayPass, identifies genetic markers subject to selection by covariates such as phenotypic or environmental variables associated with the population of interest (Gautier, 2015). This application is based on the BAYENV model proposed by Coop et al. (2010) and Günther and Coop (2013), but with several modifications detailed in Gautier (2015), including the reprogramming of the Markov chain Monte Carlo (MCMC) algorithm. For BayPass, we used a pool-size file with the –d0yij option set at 800 and 20 pilot runs with a length of 10,000 iterations, a burn-in length of 50,000 iterations, and a chain length of 50,000 iterations. For each SNP, BayPass generates a Bayes factor (BF), quantifying evidence against the null hypothesis, and an empirical Bayesian *p*-value (eBPis), a metric measuring the difference between observed data and a simulated set of data (posterior distribution) (Kass and Raftery, 1995; Andraszewicz et al., 2015; Gautier, 2015). To be considered under selection, a SNP had to meet two criteria: a BF greater than 10 (BF > 10) and an eBPis lower than 0.05 (eBPis < 0.05).

The second software, BayeScan, uses a Bayesian likelihood method that assumes a Dirichlet distribution of allele frequencies between populations (Foll, 2012). This program estimates the probability that each locus is subject to selection by using a logistic regression on the two locus-population F_ST_ coefficients. This Bayesian method uses a reversible-jump MCMC algorithm to calculate a posterior probability that each locus is under selection. The decision criterion to determine whether a locus is likely to be under a strong selection is the *q*-value (Foll, 2012), analog to a false discovery rate (FDR) *p*-value, that must be under 0.05. A second decision criterion was applied to further endorse the selected outliers: the ratio of posterior probabilities (PO). The PO threshold to affirm that a locus was under selection, in comparison with a neutral model, was set to 0.91, which corresponds to a strong relationship on the Jeffreys scale (Foll, 2012). We used BayeScan with the default parameters, but we set the minimum number of iterations to 50,000, the length of 20 pilot runs to 10,000 iterations, and the burn-in length to 50,000 iterations.

The third software, BayeScEnv, is similar to BayeScan and uses the F_ST_ index to detect loci with a high level of differentiation in comparison with the entire genome. This program allows a normalization vector to be applied to the environmental data instead of only a binary combination, thus generating a lower number of false positives, according to the creators of the software (de Villemereuil and Gaggiotti, 2015). We used BayeScEnv with the default parameters, with the number of iterations set to 50,000 and 20 pilot runs with a length of 10,000 iterations. We used the reported *q*-value, which is related to the FDR, as our decision criterion, considering only SNPs with a *q*-value less than 0.05.

### Localization of outlier loci, gene function, and genic environment

The SNPs identified in short reads were first retrieved from a draft SCN genome available from SCNBase (https://www.scnbase.org/) by means of BLASTN with the default parameters, except for a smaller word size of 4, with the Blast2GO application (Conesa et al., 2005). Many of the identified SNP-containing fragments matched multiple genes or genome locations (see Supplementary Tables 3–5). To assign a putative gene function to each SNP, we compared the aligned sequences to the National Center for Biotechnology Information (NCBI) protein database by means of BLASTX and BLASTP (Altschul et al., 1990) on a subset of sequences (nematodes, taxid: 6231) or to all of the NCBI non-redundant (nr) sequence database with an *E*-value significance cutoff of 1e^−5^.

As our GBS sequencing covers approximately 0.8% of the genome (see Results section), we explored the genomic regions around outlier loci for genetic hitchhiking in whole-genome sequences from four populations of different origin and distinct virulence profile (ON1, ON34, IL4, and KS2). Sequencing libraries were prepared using the Nextera XT DNA Library Preparation Kit (Illumina, San Diego, CA) and sequenced on a MiSeq sequencer (Illumina) using the MiSeq Reagent Kit v3 (600-cycle). Reads were demultiplexed using the Sabre software program (https://github.com/najoshi/sabre) and processed with Trimmomatic (v0.32) (Bolger et al., 2014) to remove adapters and barcodes. Alignment on the draft reference genome (see above) was done using SAMtools (v0.1.19) (Li et al., 2009) and BWA (v0.7.12) (Li and Durbin, 2009). Only a window of ± 50 kb around the 15 loci under selection was retained for the genic environment analysis. Variants were called with freebayes (v1.0.2) (Garrison and Marth, 2012) and snpeff (v4.2) (Cingolani et al., 2012). Distribution of allele frequencies in these four populations for each gene variant was compared with the allele frequency of the corresponding loci under selection in the same population in the genotyping-by-sequencing dataset. Only SNPs exhibiting a similar allele frequency and having a high impact on the predicted protein product were retained in our analysis.

## Results

### Genotyping-by-sequencing

A total of 192,576,709 short reads were obtained from the sequencing of the DNA from the 64 SCN populations, following digestion by the PstI/MspI restriction enzymes. The *H. glycines* effective genome length was estimated to be 96,752,286 bp, with an average of one PstI restriction site every 12,688 bases (E. Lord, private communication, 2017). On the basis of the sequence size used by the UNEAK (Universal Network-Enabled Analysis Kit) pipeline (first 64 bp of each read), the expected horizontal coverage was approximately 0.8% of the total SCN genome, and the vertical coverage at each locus, considering the number of reads obtained, was 400 ×. Before filtering, the UNEAK pipeline identified 3,172 variants. After filtering for low coverage and selecting only SNP variants, two datasets were generated. The first dataset contained 245 SNPs without missing data (loci sequenced in all populations), and the second contained 804 SNPs with missing data (Sequences and coverage in Supplementary Table 7).

### Population structure

#### Principal component analysis

A principal component analysis (PCA) was performed using the dataset without missing data, which contained 245 SNPs. The PCA plot of the 64 North American SCN populations revealed a geographically ordered pattern (Figure 1A). Overall, the Ontario populations showed the greatest dispersion, indicating they are more genetically differentiated, while the central regions of the US showed more clustered populations. Also, the group containing populations from Minnesota and North Dakota (in green in Figure 1A) was clearly different from the Ontario populations by the first axis, which explained 21% of the total variation. All populations from the central states, although they originated from many states and covered a much wider area, were less diverse and more clustered together.

#### Phylogenetic tree

To better understand the population structure of each population, we used the 804-SNP dataset to conduct a phylogenetic analysis based on Provesti's distance. The inferred neighbor-joining phylogenetic tree (Figure 1B) showed that most of the Ontario samples (in red in Figure 1B) were different from the rest of the North American populations. Some samples from Ontario were found to be different from each others and more similar to those of central states. Such a pattern can also be observed in the PCA analysis (Figure 1A).

#### Genetic differentiation

Overall, we observed an average value for Wright's fixation index (F_ST_) of 0.15 ± 0.07. However, pairwise genetic distance was highly variable, with a minimum F_ST_ of 0.005 and a maximum F_ST_ of 0.53 (Supplementary Table 1). The highest F_ST_ value was found between two of the most remote populations, ND6 from North Dakota and ON41 from Ontario, which were separately by approximately 1,720 km. On the other hand, the lowest F_ST_ value was observed between ON3 and ON5, which were separated by only 17.9 km. In general, higher F_ST_ values were found in distant populations, especially between populations from Minnesota (MN4 and MN5), North Dakota (ND3 and ND6), South Dakota (SD2), and Ontario (ON6, ON13, ON25, ON41, and ON46).

#### Isolation by distance

The analysis of the relationship between genetic distance [F_ST_/(1–F_ST_)] and geographic distance (km) for all pairs of populations revealed a significant (*p* = 0.012) but weak effect of isolation by distance, with a Mantel correlation (*r*) of only 0.135. However, when analyzing the effect of isolation by distance at the local scale, a different pattern was found. With respect to US states with recent SCN history, a strong and significant positive correlation was observed between the genetic and geographic distances in North Dakota (*r* = 0.430, *p* = 0.013) and Minnesota (*r* = 0.730, *p* = 0.006). In contrast, no correlation was found among the populations from Illinois (*r* = −0.170, *p* = 0.767).

**Figure 2:**
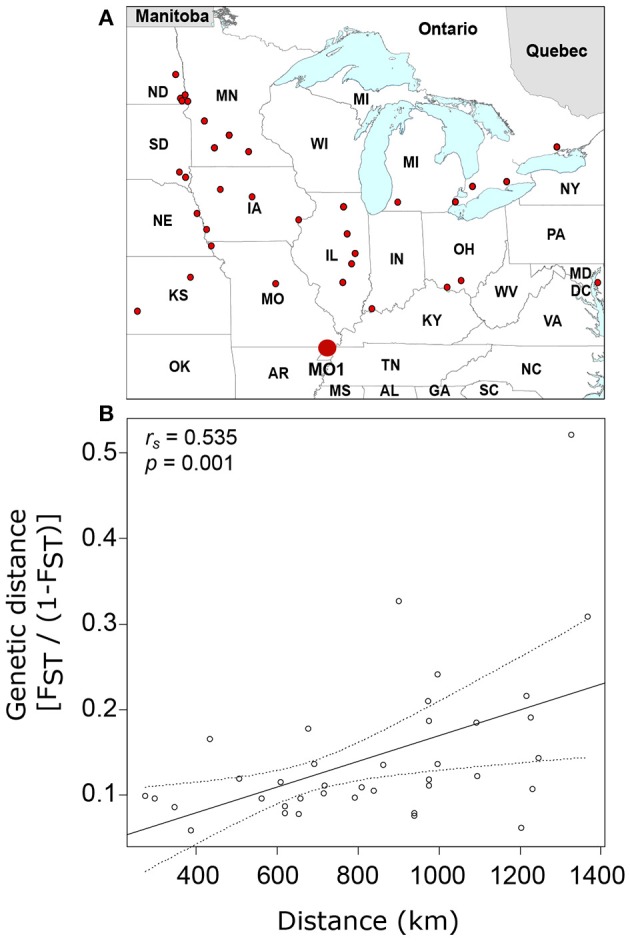
Distribution of soybean cyst nematode populations used in this study (small dots) and identification of the “oldest” sample in the dataset (MO1, large dot) **(A)** as well as the relationship between genetic distance [F_ST_/(1–F_ST_)] and geographic distance **(B)**. Linear regression line is shown with 95% confidence intervals estimated using 1,000 bootstrap replicates.

To further explore the contribution of isolation by distance to the genetic differentiation of United States and Canada populations of SCN, we compared the genetic distance of each population from a theoretical ancestor population (MO1) using the 245-SNP dataset. This analysis included 38 independent populations that were separated by 273 to 1,367 km from MO1 (Figure 2A). The genetic-distance ratio to the “oldest population” varied from 0.06 for IL1 (386 km apart) to 0.52 for ND6 (1,327 km apart) (Supplementary Table 2) and an overall moderate (*r*_*s*_ = 0.535) but significant (*p* = 0.001) relationship was observed between genetic distance and geographic distance (Figure 2B).

### Signature of genetic adaptations

#### Genome scans

**Figure 3:**
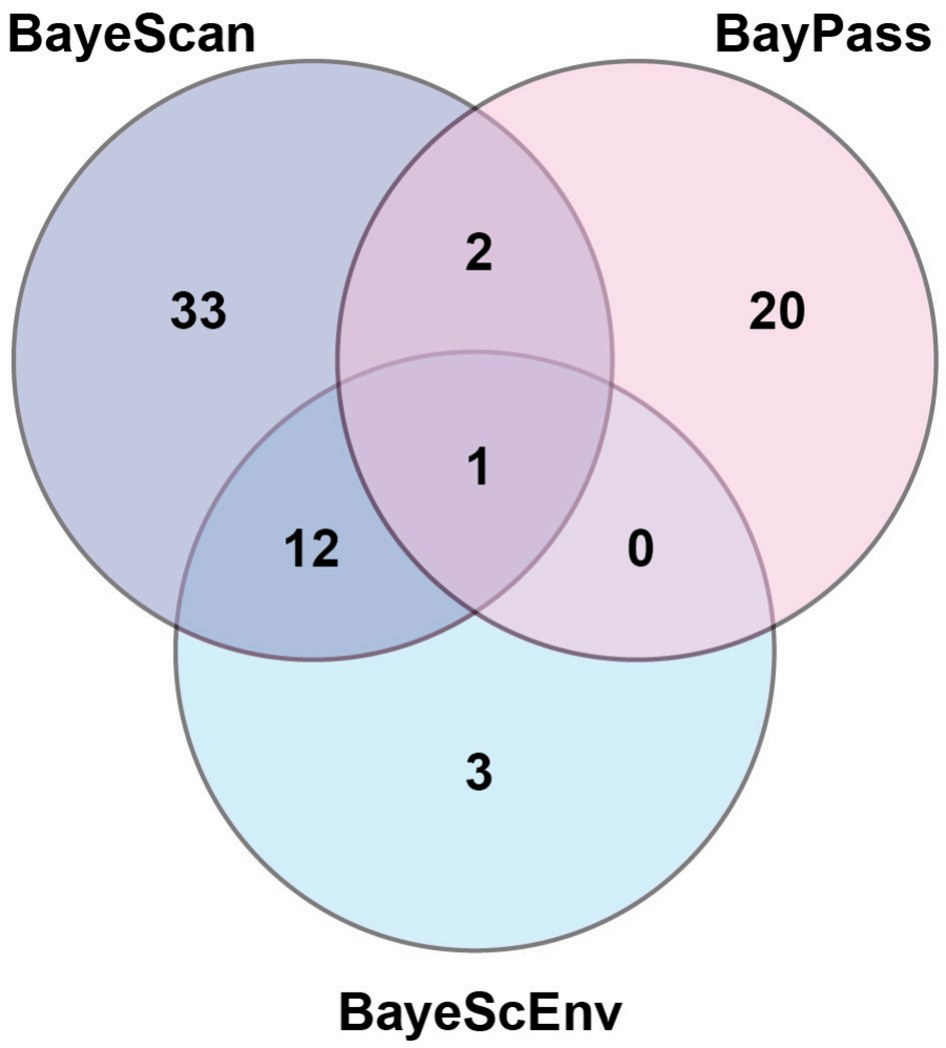
Number of outlier loci identified by three different Bayesian genome scan methods in 64 populations of the soybean cyst nematode from North America.

**Figure 4:**
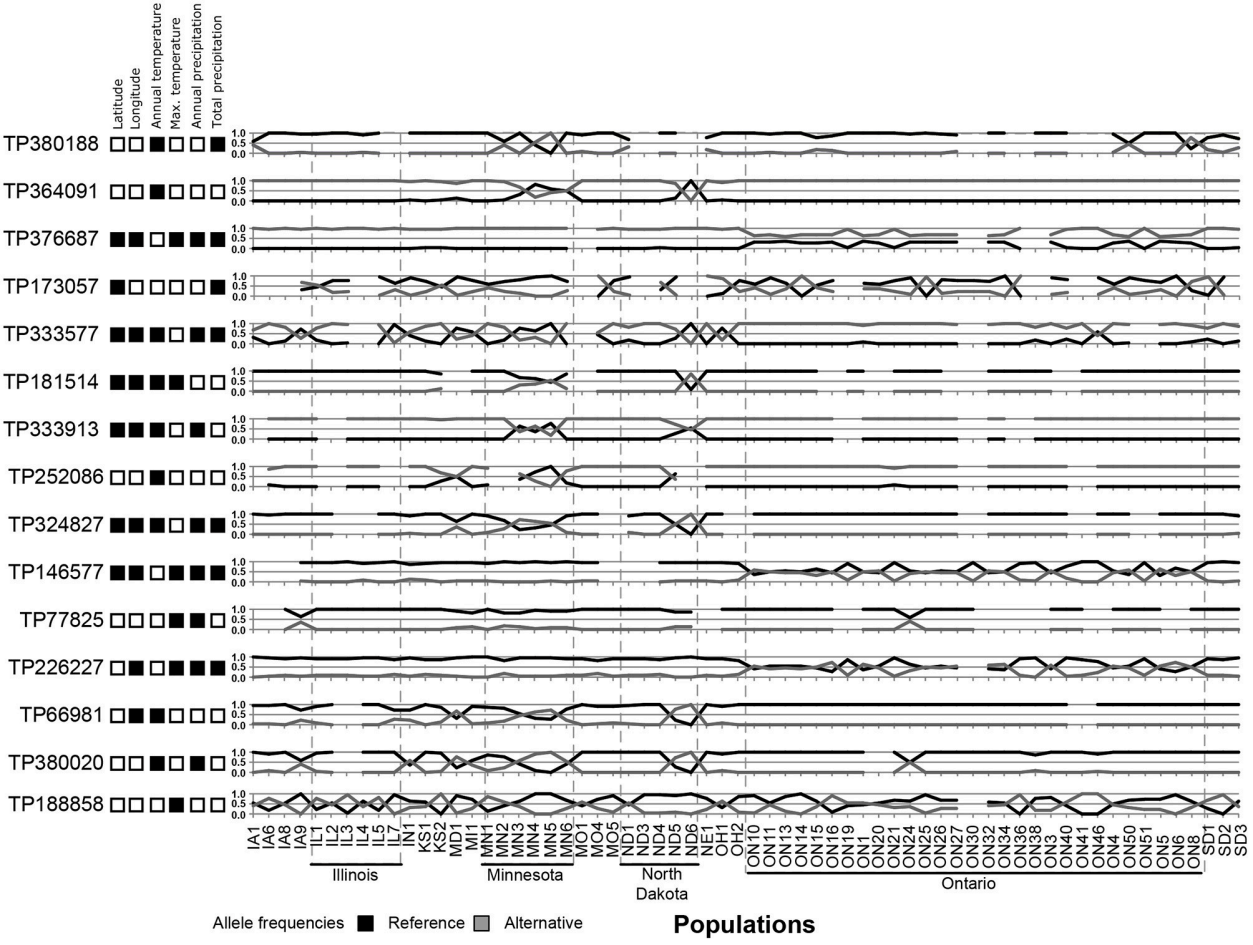
Allele frequencies of 15 outlier loci identified in North American populations of the soybean cyst nematode and associations of the loci with geographic or climatic co-variables. Black squares represent a significant association.

Using three Bayesian inference genome scan approaches, we identified 71 SNPs (out of 804) that were under selection. Among the different computational methods, 48 loci were identified by BayeScan, 23 loci were identified by BayPass, and 16 loci were identified by BayeScEnv (Supplementary Tables 3–5). Out of those 71 SNPs, only one was highlighted by the three methods, while 15 outliers were inferred by at least two of the methods (Figure 3). The geographic variables (LAT and LONG) were associated with seven and eight outlier loci, respectively, and the climatic variables (BIO1, BIO5, BIO12, and BIO18) were associated with nine, six, eight, and seven SNPs, respectively (Table 1). Three SNPs were strictly associated with only one environmental variable: TP188858 was associated with maximum temperature, while TP252086 and TP364091 appeared to be linked to annual temperature. The allele frequencies analysis at these 15 loci revealed SNPs that were specific for a given region, such as TP364091, TP181514, and TP333913 for Minnesota and North Dakota and TP376687, TP146577, and TP226227 for Ontario (Figure 4).

**Table 1:** Association of outlier loci [single-nucleotide polymorphisms (SNPs)] identified by at least two Bayesian genome scan methods in 64 populations of the soybean cyst nematode from North America with geographic or climatic co-variables.

**SNP ID**	**Method**	**Latitude**	**Longitude**	**Temperature (annual)**	**Temperature (maximum)**	**Precipitation (annual)**	**Precipitation (warmest quarter)**
TP66981	BayeScan		0.998	0.995			
	BayeScEnv		0.017	0.042			
	BayPass						
TP77825	BayeScan				0.999	0.941	
	BayeScEnv				0.029		
	BayPass						
TP146577	BayeScan	0.952	1.000		1.000	0.997	0.997
	BayeScEnv		0.047		0.038		
	BayPass						
TP173057	BayeScan	0.988					
	BayeScEnv						
	BayPass						13.86
TP181514	BayeScan	0.996	0.940	0.992	0.961		
	BayeScEnv		0.050	0.009			
	BayPass						
TP188858	BayeScan				0.999		
	BayeScEnv				0.050		
	BayPass						
TP226227	BayeScan		1.000		1.000	0.956	0.999
	BayeScEnv				0.044		
	BayPass						
TP252086	BayeScan			0.963			
	BayeScEnv			0.050			
	BayPass						
TP324827	BayeScan	0.999	0.999	0.999		1.000	0.999
	BayeScEnv	0.043	0.023	0.006		0.017	
	BayPass						
TP333577	BayeScan	0.984	0.978			0.999	1.000
	BayeScEnv		0.029	0.015			0.040
	BayPass						14.32
TP333913	BayeScan	0.931	0.978	0.976		0.930	
	BayeScEnv		0.029	0.015			
	BayPass						
TP364091	BayeScan			0.976			
	BayeScEnv			0.020			
	BayPass						
TP376687	BayeScan	0.993	0.999		1.000	0.999	0.999
	BayeScEnv				0.038		
	BayPass						
TP380020	BayeScan			0.998		1.000	
	BayeScEnv			0.030		0.027	
	BayPass						
TP380188	BayeScan			0.968			
	BayeScEnv						
	BayPass						13.97

#### Localization, effect, and genic environment of outlier loci

Of the 15 SNPs under selection identified by at least two Bayesian approaches, 10 SNPs were located in predicted genes (seven in exons and three in introns), and five SNPs were located in intergenic regions (Table 2). Two SNPs (TP380188 and TP364091) correspond to non-synonymous mutations that induce a change in the amino acid sequence of the resulting protein. The annotations of the genes putatively impacted by these modifications are SMAD-4 (mothers against decapentaplegic homolog 4) for TP380188 and DOP-3 (dopamine receptor 3) for TP364091. The sequence corresponding to TP364091 was retrieved in four different genes (g14639.t1, g14642.t1, g14644.t1, and g14654.t1) in the draft reference genome and introduced two different mutations (Supplementary Tables 3–5). All the other SNPs (13/15) were silent mutations (synonymous in exons or located in introns or intergenic regions) and thus probably not the cause of their selection. Among the 42 remaining SNPs that were under selection but identified by only one pipeline, 12 correspond to a non-synonymous modification impacting the predicted protein sequence. These SNPs include the gene UFM1 (Ubiquitin-fold modifier 1), the gene PLA2 (85/88 kDa calcium-independent phospholipase A2), and a pyruvate kinase (Supplementary Tables 3–5).

**Table 2:** Identification and effect of outlier loci [single-nucleotide polymorphisms (SNPs)] identified by at least two Bayesian genome scan methods in 64 populations of the soybean cyst nematode from North America.

**ID**	**Location**	**S or N-S^1^**	**SNP**	**a.a. subst.^2^**	***E*-value**	**Best annotation**
TP380188	Exon	N-S	C/A	Gln/Lys	2.0e-20	SMAD-4 [*Strongyloides ratti*]
TP364091	Exon	N-S	G/A	Asp/Asn	2.6e-104	Dopamine receptor 3 [*Toxocara canis*]
TP376687	Exon	S	T/G	Gly/Gly	4.0e-11	Putative esophageal gland cell protein Hgg-20 [*Heterodera glycines*]
TP173057	Exon	S	C/T	Ile/Ile	2.0e-11	Leucine-rich repeat-containing domain protein, partial [*Necator americanus*]
TP333577	Exon	S	C/T	Leu/Leu	1.0e-110	Cadherin-4 [*Caenorhabditis elegans*]
TP181514	Exon	S	T/C	Gly/Gly	1.6e-100	LET-19 [*Caenorhabditis brenneri*]
TP333913	Exon	S	T/C	Gly/Gly	1.6e-7	EGL-17 [*Brugia malayi*]
TP252086	Intron	–	G/A	–	2.0e-64	Esophageal gland-localized secretory protein 11 [*Heterodera glycines*]
TP324827	Intron	–	C/T	–	3.0e-32	Reverse transcriptase [*Ancylostoma ceylanicum*]
TP146577	Intron	–	T/A	–	2.0e-11	Hypothetical protein Y032_0209g2110 [*Ancylostoma ceylanicum*]
TP77825	Intergenic	–	T/A	–	2.0e-57	Nearest gene: hypothetical protein CRE_19487 [*Caenorhabditis remanei*]
TP226227	Intergenic	–	T/G	–	3.0e-05	Nearest gene: ATP-dependent DNA helicase PIF1, partial [*Toxocara canis*]
TP66981	Intergenic	–	T/G	–	–	*No significant hit*
TP380020	Intergenic	–	G/T	–	–	*No significant hit*
TP188858	Intergenic	–	G/A	–	–	*No significant hit*

1 *Synonymous (S) or non-synonymous (N-S) mutations in the final protein product*.

2 *a.a., amino acid substitution*.

The whole-genome resequencing of four SCN populations for the exploration of the genic environment of outlier loci highlighted 257 genes containing high-impact variants in a ± 50-kb window around loci under selection. Annotation was available for 148 of these genes, corresponding to 132 different gene functions (Supplementary Table 6). Of these genes, 25 exhibited allele frequencies similar to their neighboring outlier loci (Table 3).

**Table 3:** Putative hitchhiking gene variants with high-impact neighboring loci under selection and exhibiting a similar allele frequency in *Heterodera glycines*.

**ID**	**Gene**	**Best annotation**	***E*-value^1^**	**Type of variants^2^**
TP146577	g15502.t1	G-protein-coupled receptor, partial [*Pristionchus pacificus*]	9.10e-57	complex, insertion
TP173057	g18365.t1	85 kda calcium-independent phospholipase a2 [*Ascaris suum*]	8.50e-46	SNP, insertion
TP173057	g18358.t2	F-box only protein 30 [*Ascaris suum*]	1.10e-33	4 complexes
TP173057	g18327.t1	Protein CBR-CLEC-223 [*Caenorhabditis briggsae*]	5.70e-57	**SNP**
TP173057	g18328.t1	Protein lap1 [*Toxocara canis*]	1.80e-104	6 SNP, 3 insertions, 2 complexes
TP173057	g18332.t1	Protein scribble-like protein, partial [*Stegodyphus mimosarum*]	1.00e-33	2 deletions, **MNP**
TP181514	g3633.t1	Mediator of RNA polymerase II transcription subunit 13-like protein [*Ascaris suum*]	1.20e-133	Deletion
TP226227	g1982.t1	85/88 kDa calcium-independent phospholipase A2 [*Toxocara canis*]	8.00e-29	**SNP**
TP226227	g14545.t1	Glycoside hydrolase domain containing protein [*Haemonchus contortus*]	1.10e-162	deletion, deletion
TP226227	g12205.t1	Kazal domain and organic anion transporter polypeptide (OATP) family and MFS [*Strongyloides ratti*]	3.00e-36	deletion, 3 complexes
TP226227	g1979.t1	Serine/threonine-protein kinase haspin [*Haemonchus contortus*]	1.40e-62	insertion
TP252086	g18812.t1	Bestrophin/UPF0187 family-containing protein [*Strongyloides ratti*]	2.30e-44	deletion
TP252086	g18825.t1	mtN3/saliva family protein [*Oesophagostomum dentatum*]	1.30e-55	deletion, SNP, complex
TP333577	g16696.t2	AFG3-like protein 2 [*Toxocara canis*]	1.30e-286	complex, 2 insertions
TP333913	g14633.t1	Cytohesin-1 [*Trichinella zimbabwensis*]	1.30e-118	SNP
TP333913	g14626.t1	Fibroblast growth factor 17 [*Toxocara canis*]		deletion
TP333913	g14617.t1	Immunoglobulin domain containing protein [*Haemonchus contortus*]	9.60e-42	deletion
TP333913	g14614.t1	Sodium-dependent acetylcholine transporter [*Toxocara canis*]	1.50e-215	SNP
TP364091	g14633.t1	Cytohesin-1 [*Trichinella zimbabwensis*]	1.30e-118	SNP
TP364091	g14656.t1	Major facilitator superfamily MFS-1 domain containing protein, partial [*Haemonchus contortus*]	2.00e-106	deletion
TP380020	g7291.t1	Carboxypeptidase [*Eupolyphaga sinensis*]	1.00e-60	**SNP**
TP380020	g8313.t1	Cell division cycle related [*Caenorhabditis elegans*]	1.10e-254	deletion, SNP
TP380020	g8311.t1	GPI ethanolamine phosphate transferase 3, partial [*Trichinella papuae*]	1.90e-125	deletion
TP380020	g8314.t3	Homeobox protein ceh-6 [*Toxocara canis*]	4.60e-52	insertion, 2 deletions
TP380020	g8309.t1	Immunoglobulin I-set domain containing protein [*Ascaris suum*]	5.50e-31	2 deletions
TP380020	g8305.t1	RecQ-mediated genome instability protein 1 [*Toxocara canis*]	4.60e-28	**SNP**
TP380188	g2288.t1	Cleavage polyadenylation specificity factor domain containing protein [*Haemonchus contortus*]	3.90e-209	insertion
TP380188	g2287.t1	Cytochrome b-c1 complex subunit 2, mitochondrial [*Strongyloides ratti*]	1.20e-63	deletion, SNP

1 *Expected value for annotations of each putative hitchhiking gene*.

2 *Variant in bold indicates a stop codon gain. More details on types of variants and position in the gene are provided in Supplementary Table 6. SNP, single-nucleotide polymorphism; MNP, multiple-nucleotide polymorphism*.

## Discussion

Since its introduction into North America in 1954, the soybean cyst nematode has spread at a regular pace to most of the soybean-producing areas in the US and reached Canada in 1987. Its northern limit of establishment remains to be determined, as new populations are found each year. Soybean acreage is rapidly increasing in northern latitudes; for example, farmers in the province of Manitoba, Canada, sowed 2.3 million acres with soybean in 2017, a 40% increase in 1 year in a province where soybean was not grown a decade ago (Statistics Canada, 2017). This spectacular change in land use results from a coordinated breeding program for short-season and drought-tolerant soybean cultivars (Tardivel et al., 2014). Although soybean is nowadays very profitable in Canada (Manitoba, Northern Ontario, Québec), managing a pest such as SCN could be challenging, as no resistant cultivars are currently adapted to these regions. Simulations based on thermal development have shown that SCN could theoretically survive in these new soybean growing areas (Gendron St-Marseille, 2013), but actual establishment is a different matter, as initial observations in the province of Québec indicated that even though the nematode is detected in many regions, populations weakly reproduced (Mimee et al., 2016). This phenomenon probably reflects SCN's very recent history in this part of the world and the poor fitness of the introduced populations but does not exclude possible adaptations of the nematode in the future. To better understand the evolution of SCN population genetics in North America, we compared 64 populations originating from 11 US states and the province of Ontario in Canada.

The dynamics of cyst nematode populations are intrinsically complex. Several thousand cysts from different females can form on a single plant, and each cyst contains hundreds of half-sibling individuals as a result of SCN's polyandrous mode of reproduction. Thus, the number of unique genotypes occurring in a field is massive. Isolating individual nematodes, even hundreds of them, to explore the genetic relationship between populations would be an arduous task and the approach would still be biased owing to limited sub-sampling. For these reasons, we opted for a Pool-Seq (sequencing of pooled DNA samples) approach. Sequencing DNA from pooled samples for each population also has the advantage of keeping the number of redundant sequences low (Schlötterer et al., 2014). However, this approach does not allow the assignment of sequences to individuals and prevents some population genetic analyses such as linkage-disequilibrium estimation (Lynch et al., 2014; Anand et al., 2016). Nevertheless, a number of recent studies have overcome these limitations and have proposed new methods to analyze genetic variations among populations based on Pool-Seq data (Van Tassell et al., 2008; Gautier et al., 2013; Navon et al., 2013; Mimee et al., 2015; Anand et al., 2016).

Both the PCA and phylogenetic analyses showed strong clustering of populations based on their geographic origin, which supports the “nearest-neighbor theory” movement as in the stepping-stone model (Kimura and Weiss, 1964; Hutchison and Templeton, 1999). We observed a geographic separation dividing SCN populations into two clusters, a northeastern one (Ontario) and a northwestern one (Minnesota, North Dakota) that both share genetic similarities with central populations. In northeast and northwest regions, SCN populations established only recently, and new detections continue to occur in the northern areas. In the central states, SCN populations have been present for a longer time and resistant soybean cultivars are routinely used. We believed that these two conditions contribute to explain the homogeneity of the central populations. On one hand, the continuous exchange of genetic material would have led to the homogenization of alleles in sympatric populations. On the other hand, the massive use of nematode resistant cultivars that are all derived from a single source of resistance (PI 88788) would have selected for the virulent SCN genotypes, thereby limiting the propagation of some alleles. It is thus surprising to observe a greater genetic differentiation in SCN populations from Ontario since the main assumption is that they originate from central states. These populations should contain less diversity, owing to the founder effect, which would normally result in reduced potential for differentiation. However, the selective pressures mentioned above are not yet present in Ontario, allowing the development of most genotypes. This finding concurs with the results of Faghihi et al. (2010), who observed a much greater diversity in SCN population phenotypes (HG types) in Ontario than in Tennessee, Indiana, or Illinois. A similar pattern was obtained in Minnesota by Zheng et al. (2006) and in China by Liu et al. (1997) when comparing populations from the north to populations from central regions. However, all these authors suggested that climatic conditions or local environmental factors may also play a role.

According to Hartl and Clark (2007), the overall average F_ST_ value of 0.15 that we obtained by comparing all pairs of SCN populations from North America corresponds to a high level of differentiation. However, when we examined specific pairs of populations, we found that the populations were very similar at the local scale. Also, the differentiation of recent populations (Ontario, Minnesota, North Dakota) from those of the central states was only moderate, whereas comparing these recent populations together generated very high F_ST_ values (up to 0.53). This corroborates results from the PCA analysis and clearly suggests that populations diverged following two distinct routes, one in the northwest toward North Dakota and one in the northeast toward Ontario. This hypothesis is further supported by the isolation-by-distance analysis. Overall, we observed a significant effect of distance on genetic differentiation at the continental scale. The effect of distance was even more noticeable in newly colonized areas like North Dakota and Minnesota or when each population was compared to a common ancestor. On the other hand, the absence of any effect of distance in Illinois and low F_ST_ in the central states clearly suggests that dispersal is frequent enough to weaken signs of isolation by distance after a short period of time. This continuous gene flow combined with the selection of alleles due to the general use of the same resistant soybean lines are thought to cause a rapid homogenization of SCN populations (Mitchum et al., 2007; Niblack et al., 2008; Zheng and Chen, 2011). If SCN is already adapted to environmental conditions prevailing in northern areas, this homogenization process would accelerate the development of virulent genotypes in these regions (in comparison with the central states) and would be independent from the use of resistant cultivars. This concurs with the finding of Faghihi et al. (2010) where 15% of SCN populations in Ontario were able to multiply on PI 548402 and 6% were able to multiply on PI 90763, even though these sources of resistance were not present in commercial cultivars in Ontario.

Although 71 SNPs were found to be under selection among SCN populations, only one SNP was identified by the three Bayesian approaches tested. This SNP (TP333577) was strongly associated with precipitation; but the geographic distribution of allele frequencies (Figure 4) at that specific locus did not match with the general pattern of genetic differentiation, instead showing a very local effect (in populations from Minnesota but also in some populations from Iowa, Illinois, North Dakota, and Ohio). Thus, this selection for wetness was either lost in favor of a more stringent pressure or too costly to be maintained in the other regions. Overall, considering the broad range of environmental conditions in which these populations develop, finding only a few genes under selection suggests that SCN possesses an intrinsic capacity to evolve within a large range of temperature and wetness gradients.

Most of the 15 outliers identified by at least two pipelines correlated with temperature, reinforcing that temperature is a key factor that acts on the genetic diversity and natural selection of nematode populations. Nematodes are poikilothermic organisms and depend greatly on temperature to complete their life cycle. Thus, any adaptation that facilitates the development and reproduction of nematodes at lower temperatures would be advantageous. On the basis of the distribution of allele frequencies, however, this adaptation does not seem crucial for survival, as the northern populations did not all exhibit similar patterns. This trait is probably not yet fixed in these populations. Nevertheless, six SNPs under selection and associated with temperature were specific to populations from Ontario (TP376687, TP146577, and TP226227) or only retrieved from Minnesota and North Dakota (TP364091, TP181514, and TP333913), a finding that clearly suggests local adaptations.

The SNP corresponding to TP364091 was retrieved in four out of six populations from Minnesota and in one population from North Dakota. That SNP, which is a non-synonymous mutation in the gene DOP-3 (dopamine receptor 3) that replaces an aspartic acid with an asparagine, was strongly associated with the annual mean temperature and annual precipitation. This modification was retrieved in two distinct gene sequences (g14642.t1 and g14654.t1), and a synonymous mutation (Pro/Pro) was retrieved in two other sequences (g14639.t1 and g14644.t1) but in a different amino acid, indicating a reading frame shift and a putative upstream indel. These four genes were all annotated as DOP-3 and were probably the result of gene duplication. Gene duplication has frequently been hypothesized to play an important role in adaptation to the environment (reviewed in Kondrashov, 2012). In *Caenorhabditis elegans*, the *DOP-3* protein is well known to modulate chemosensory functions, such as mating and foraging, and to be involved in locomotion (Chase et al., 2004; Wood and Ferkey, 2016). The other SNP that introduces a non-synonymous mutation and was highlighted by two genome scan methods, namely, TP380188, was located in the gene SMAD-4 (mothers against decapentaplegic homolog 4) and was retrieved in only a few populations from Minnesota, North Dakota, and Ontario. The *SMAD-4* protein is a regulator of different cellular processes, including cell differentiation, apoptosis, migration, and proliferation (Nikolic et al., 2011). Mutations of *SMAD-2, SMAD-3, SMAD-4*, or *SMAD-6* were previously shown to result in a 30% reduction in body size in *C. elegans* (Savage-Dunn et al., 2003; Watanabe et al., 2007). Also of interest is the locus TP122360, identified only by BayPass, that was located in the UFM1 (ubiquitin-fold modifier 1) genes (g7250.t1 and g7228.t1). A deletion in this gene in *C. elegans*, although reducing reproduction rate and life span, increased the survival of this nematode under oxidative or heat stress (Hertel et al., 2013).

For the majority of the loci, their selection probably results from genetic hitchhiking rather than a direct contribution to adaptation. To explore that possibility, we analyzed the genic environment in a ± 50 kb window around each SNP in four SCN populations. Genes containing high-impact genetic variations and exhibiting the same allele frequencies as the associated SNP under selection were of particular interest. Two of these genes (g18327.t1 and g15502.t1) coded for G-protein-coupled receptors (GPCR). These proteins are members of a large and very diverse multigene family with hundreds of occurrences in the *C. elegans* genome (Bockaert and Pin, 1999; Bargmann, 2006). The GCPR are crucial in sensing the local environment and were shown to evolve following alterations in habitat or foraging behavior (Nei et al., 2008). Four genes coding for proteins implicated in metabolite transport into the cell were also identified (g12205.t1, g14656.t1, g18812.t1, and g18825.t1), as was one gene involved in the regulation of those proteins (g18365.t1). All these proteins are required to maintain homeostasis in the cell and to respond to local environmental changes. In a similar study using the fungus *Fagus sylvatica*, Pluess et al. (2016) found that a version of a potassium transporter was associated with lower precipitation and could contribute to the regulation of growth under dry conditions. Structural changes in the body of an organism as a result of microevolution can also confer a significant advantage in terms of resisting more adverse environmental conditions, such as drought or high temperatures (Hazel and Williams, 1990). In our study, mutations were observed in two genes involved in cell-membrane and cell-wall stability (g18328.t1 and g8311.t1). Lastly, modifications in genes involved in the regulation of transcription (g2288.t1 and g3633.t1) and in the maturation of proteins (g14633.t1 and g18358.t2) have the potential to radically change the proteome of the adapted organism.

Although some SCN genes were found to be under selection and local adaptation was found to be underway in this study, our results also indicate that there is no critical adaptive event required for SCN establishment in northern latitudes. Consequently, all populations should theoretically survive and multiply at high latitudes. The risk is thus real for new soybean areas where cultivars resistant to SCN are not available at this time. Of course, the method we used does not explore the entire genome, and a pan-genomic study of these populations could reveal other loci under selection in an evolutionary process.

## Data availability statement

The raw data supporting the conclusions of this manuscript will be made available by the authors, without undue reservation, to any qualified researcher.

## Author contributions

BM, A-FG, and JB conceived and designed the experiments. A-FG, EL, and P-YV performed the experiments and analyzed the data. A-FG, EL, and BM wrote the paper. All the authors revised the manuscript.

### Conflict of interest statement

The authors declare that the research was conducted in the absence of any commercial or financial relationships that could be construed as a potential conflict of interest.

## References

[B1] Altschul S. F. Gish W. Miller W. Myers E. W. Lipman D. J. (1990). Basic local alignment searchtool. J. Mol. Biol. 215, 403–410. 10.1016/S0022-2836(05)80360-2 2231712

[B2] Anand S. Mangano E. Barizzone N. Bordoni R. Sorosina M. Clarelli F.. (2016). Next generation sequencing of pooled samples: guideline for variants' filtering. Sci. Rep. 6:33735. 10.1038/srep33735 27670852 PMC5037392

[B3] Anderson T. R. Welacky T. W. Olechowski H. T. Ablett G. Ebsary B. A. (1988). First report of *Heterodera glycines* on soybeans in Ontario, Canada. Plant Dis. 72:453 10.1094/PD-72-0453C

[B4] Andraszewicz S. Scheibehenne B. Rieskamp J. Grasman R. Verhagen J. Wagenmakers E.-J. (2015). An introduction to Bayesian hypothesis testing for management research. J. Manag. 41, 521–543. 10.1177/0149206314560412

[B5] Bargmann C. I. (2006). Comparative chemosensation from receptors to ecology. Nature 444, 295–301. 10.1038/nature05402 17108953

[B6] Blok V. C. Phillips M. S. Harrower B. E. (1997). Comparison of British populations of potato cyst nematodes with populations from continental Europe and South America using RAPDs. Genome 40, 286–293. 10.1139/g97-040 18464829

[B7] Bockaert J. Pin J. P. (1999). Molecular tinkering of G protein-coupled receptors: an evolutionary success. EMBO J. 18, 1723–1729. 10.1093/emboj/18.7.1723 10202136 PMC1171258

[B8] Bolger A. M. Lohse M. Usadel B. (2014). Trimmomatic: a flexible trimmer for illumina sequence data. Bioinformatics 30, 2114–2120. 10.1093/bioinformatics/btu170 24695404 PMC4103590

[B9] Boucher A. C. Mimee B. Montarry J. Bardou-Valette S. Bélair G. Moffett P.. (2013). Genetic diversity of the golden potato cyst nematode *Globodera rostochiensis* and determination of the origin of populations in Quebec, Canada. Mol. Phylogenet. Evol. 69, 75–82. 10.1016/j.ympev.2013.05.020 23742887

[B10] Bradbury P. J. Zhang Z. Kroon D. E. Casstevens T. M. Ramdoss Y. Buckler E. S. (2007). TASSEL: software for association mapping of complex traits in diverse samples. Bioinformatics 23, 2633–2635. 10.1093/bioinformatics/btm308 17586829

[B11] Cariou M. Duret L. Charlat S. (2013). Is RAD-seq suitable for phylogenetic inference? An *in silico* assessment and optimization. Ecol. Evol. 3, 846–852. 10.1002/ece3.512 23610629 PMC3631399

[B12] Chase D. L. Pepper J. S. Koelle M. R. (2004). Mechanism of extrasynaptic dopamine signaling in *Caenorhabditis elegans*. Nat. Neurosci. 7, 1096–1103. 10.1038/nn1316 15378064

[B13] Cingolani P. Platts A. Wang L. L. Coon M. Nguyen T. Wang L.. (2012). A program for annotating and predicting the effects of single nucleotide polymorphisms, SnpEff: SNPs in the genome of *Drosophila melanogaster* strain w^1118^; *iso*-2; *iso*-3. Fly 6, 80–92. 10.4161/fly.19695 22728672 PMC3679285

[B14] Colgrove A. L. Niblack T. L. (2008). Correlation of female indices from virulence assays on inbred lines and field populations of *Heterodera glycines*. J. Nematol. 40, 39–45. 19259518 PMC2586527

[B15] Conesa A. Götz S. García-Gómez J. M. Terol J. Talón M. Robles M. (2005). Blast2GO: a universal tool for annotation, visualization and analysis in functional genomics research. Bioinformatics 21, 3674–3676. 10.1093/bioinformatics/bti610 16081474

[B16] Coop G. Witonsky D. Di Rienzo A. Pritchard J. K. (2010). Using environmental correlations to identify loci underlying local adaptation. Genetics 185, 1411–1423. 10.1534/genetics.110.114819 20516501 PMC2927766

[B17] Davis E. L. Tylka G. L. (2000). Soybean cyst nematode disease. Plant Health Instr. 10.1094/PHI-I-2000-0725-01

[B18] de Villemereuil P. Gaggiotti O. E. (2015). A new F_ST_-based method to uncover local adaptation using environmental variables. Meth. Ecol. Evol. 6, 1248–1258. 10.1111/2041-210X.12418

[B19] Early R. Sax D. F. (2014). Climatic niche shifts between species' native and naturalized ranges raise concern for ecological forecasts during invasions and climate change. Glob. Ecol. Biogeogr. 23, 1356–1365. 10.1111/geb.12208

[B20] Eizaguirre C. Baltazar-Soares M. (2014). Evolutionary conservation-evaluating the adaptive potential of species. Evol. Appl. 7, 963–967. 10.1111/eva.12227

[B21] Elshire R. J. Glaubitz J. C. Sun Q. Poland J. A. Kawamoto K. Buckler E. S.. (2011). A robust, simple genotyping-by-sequencing (GBS) approach for high diversity species. PLoS ONE 6:e19379. 10.1371/journal.pone.0019379 21573248 PMC3087801

[B22] Eoche-Bosy D. Gautier M. Esquibet M. Legeai F. Bretaudeau A. Bouchez O.. (2017). Genome scans on experimentally evolved populations reveal candidate regions for adaptation to plant resistance in the potato cyst nematode *Globodera pallida*. Mol. Ecol. 26, 4700–4711. 10.1111/mec.14240 28734070

[B23] Eves-van den Akker S. Lilley C. J. Reid A. Pickup J. Anderson E. Cock P. J.. (2015). A metagenetic approach to determine the diversity and distribution of cyst nematodes at the level of the country, the field and the individual. Mol. Ecol. 24, 5842–5851. 10.1111/mec.13434 26607216 PMC4981918

[B24] Faghihi J. Donald P. A. Noel G. Welacky T. W. Ferris V. R. (2010). Soybean resistance to field populations of *Heterodera glycines* in selected geographic areas. Plant Health Prog. 10.1094/PHP-2010-0426-01-RS

[B25] Fick S. E. Hijmans R. J. (2017). WorldClim 2: new 1-km spatial resolution climate surfaces for global land areas. Int. J. Climatol. 37, 4302–4315. 10.1002/joc.5086

[B26] Foll M. (2012). BayeScan v2.1 User Manual. Available online at: http://cmpg.unibe.ch/software/BayeScan/files/BayeScan2.1_manual.pdf

[B27] Foll M. Gaggiotti O. E. (2008). A genome-scan method to identify selected loci appropriate for both dominant and codominant markers: a Bayesian perspective. Genetics 180, 977–993. 10.1534/genetics.108.092221 18780740 PMC2567396

[B28] Fu Y.-B. Peterson G. W. Dong Y. (2016). Increasing genome sampling and improving SNP genotyping for genotyping-by-sequencing with new combinations of restriction enzymes. G3 6, 845–856. 10.1534/g3.115.025775 26818077 PMC4825655

[B29] Garrison E. Marth G. (2012). Haplotype-Based Variant Detection from Short-Read Sequencing. *arXiv [preprint] arXiv*:1207.3907.

[B30] Gautier M. (2015). Genome-wide scan for adaptive divergence and association with population-specific covariates. Genetics 201, 1555–1579. 10.1534/genetics.115.181453 26482796 PMC4676524

[B31] Gautier M. Gharbi K. Cezard T. Foucaud J. Kerdelhué C. Pudlo P.. (2013). The effect of RAD allele dropout on the estimation of genetic variation within and between populations. Mol. Ecol. 22, 3165–3178. 10.1111/mec.12089 23110526

[B32] Gendron St-Marseille A.-F. (2013). Le nématode à Kyste du Soja (Heterodera glycines) : Enjeux des Changements Climatiques sur sa Distribution, sa Reproduction et sur les Probabilités de Synchronisme Avec le Soja (Glycine max) au Québec. Master's Thesis, Université de Sherbrooke, Sherbrooke, QC. Available online at: http://hdl.handle.net/11143/7214

[B33] Green C. D. Greet D. N. Jones F. G. W. (1970). The influence of multiple mating on the reproduction and genetics of *Heterodera rostochiensis* and *H. schachtii*. Nematologica 16, 309–326. 10.1163/187529270X00333

[B34] Grenier E. Bossis M. Fouville D. Renault L. Mugniéry D. (2001). Molecular approaches to the taxonomic position of Peruvian potato cyst nematodes and gene pool similarities in indigenous and imported populations of *Globodera*. Heredity 86, 277–290. 10.1046/j.1365-2540.2001.00826.x 11488965

[B35] Günther T. Coop G. (2013). Robust identification of local adaptation from allele frequencies. Genetics 195, 205–220. 10.1534/genetics.113.152462 23821598 PMC3761302

[B36] Hartl D. L. Clark A. G. (2007). Principles of Population Genetics. 4th Edn. Sunderland, Massachusetts Sinauer Associates.

[B37] Hassan M. A. Pham T. H. Shi H. Zheng J. (2013). Nematodes threats to global food security. Acta. Agric. Scand. B. Soil Plant Sci. 63, 420–425. 10.1080/09064710.2013.794858

[B38] Hazel J. R. Williams E. E. (1990). The role of alterations in membrane lipid composition in enabling physiological adaptation of organisms to their physical environment. Prog. Lipid Res. 29, 167–227. 10.1016/0163-7827(90)90002-3 2131463

[B39] Hegge A. H. (1957). Soybean cyst nematode, *Heterodera glycines*, in Missouri. Plant Dis. Rep. 41:201.

[B40] Hertel P. Daniel J. Stegehake D. Vaupel H. Kailayangiri S. Gruel C.. (2013). The ubiquitin-fold modifier 1 (Ufm1) cascade of *Caenorhabditis elegans*. J. Biol. Chem. 288, 10661–10671. 10.1074/jbc.M113.458000 23449979 PMC3624446

[B41] Hulme P. E. (2009). Trade, transport and trouble: managing invasive species pathways in an era of globalization. J. Appl. Ecol. 46, 10–18. 10.1111/j.1365-2664.2008.01600.x

[B42] Hutchison D. W. Templeton A. R. (1999). Correlation of pairwise genetic and geographic distance measures: inferring the relative influences of gene flow and drift on the distribution of genetic variability. Evolution 53, 1898–1914. 10.1111/j.1558-5646.1999.tb04571.x 28565459

[B43] Kamvar Z. N. Tabima J. F. Grünwald N. J. (2014). Poppr: an R package for genetic analysis of populations with clonal, partially clonal, and/or sexual reproduction. PeerJ 2:e281 10.7717/peerj.281 24688859 PMC3961149

[B44] Kass R. E. Raftery A. E. (1995). Bayes factors. J. Am. Stat. Assoc. 90, 773–795. 10.1080/01621459.1995.10476572

[B45] Kimura M. Weiss G. H. (1964). The stepping stone model of population structure and the decrease of genetic correlation with distance. Genetics 49, 561–576. 17248204 10.1093/genetics/49.4.561 PMC1210594

[B46] Kofler R. Pandey R. V. Schlötterer C. (2011). PoPoolation2: identifying differentiation between populations using sequencing of pooled DNA samples (pool-Seq). Bioinformatics 27, 3435–3436. 10.1093/bioinformatics/btr589 22025480 PMC3232374

[B47] Kondrashov F. A. (2012). Gene duplication as a mechanism of genomic adaptation to a changing environment. Proc. R. Soc. B. 279, 5048–5057. 10.1098/rspb.2012.1108 22977152 PMC3497230

[B48] Kristjansson G. (2010). 09−59.Plant Health Risk Assessment Unit of the Science Advice Division: Canadian Food Inspection Agency. 45p.

[B49] Li H. Durbin R. (2009). Fast and accurate short read alignment with Burrows–Wheeler Transform. Bioinformatics 25, 1754–1760. 10.1093/bioinformatics/btp324 19451168 PMC2705234

[B50] Li H. Handsaker B. Wysoker A. Fennell T. Ruan J. Homer N.. (2009). The sequence alignment/map format and SAMtools. Bioinformatics 25, 2078–2079. 10.1093/bioinformatics/btp352 19505943 PMC2723002

[B51] Liu X. H. Li J. Q. Zhang D. S. (1997). History and status of soybean cyst nematode in China. Int. J. Nematol. 7, 18–25.

[B52] Lu F. Lipka A. E. Glaubitz J. Elshire R. Cherney J. H. Casler M. D.. (2013). Switchgrass genomic diversity, ploidy, and evolution: novel insights from a network-based SNP discovery protocol. PLoS Genet. 9:e1003215. 10.1371/journal.pgen.1003215 23349638 PMC3547862

[B53] Lynch M. Bost D. Wilson S. Maruki T. Harrison S. (2014). Population-genetic inference from pooled-sequencing data. Genome Biol. Evol. 6, 1210–1218. 10.1093/gbe/evu085 24787620 PMC4040993

[B54] Mathew F. Markell S. Jantzi D. Yan G. Nelson B. Helms T. (2015). Soybean cyst nematode. Plant Disease Management. Fargo, ND: North Dakota State University Extension Service), p. 4.

[B55] Mimee B. Duceppe M.-O. Véronneau P.-Y. Lafond-Lapalme J. Jean M. Belzile F.. (2015). A new method for studying population genetics of cyst nematodes based on Pool-Seq and genomewide allele frequency analysis. Mol. Ecol. Resour. 15, 1356–1365. 10.1111/1755-0998.12412 25846829

[B56] Mimee B. Gagnon A.-È. Colton-Gagnon K. Tremblay É. (2016). Portrait de la situation du nématode à kyste du soja (*Heterodera glycines*) au Québec (2013–2015). Phytoprotection 96, 33–42. 10.7202/1038941ar

[B57] Mimee B. Peng H. Popovic V. Yu Q. Duceppe M.-O. Tétreault M.-P. (2014). First report of soybean cyst nematode (*Heterodera glycines* Ichinohe) on soybean in the province of Quebec, Canada. Plant Dis. 98:429 10.1094/PDIS-07-13-0782-PDN 30708422

[B58] Mitchum M. G. Wrather J. A. Heinz R. D. Shannon J. G. Danekas G. (2007). Variability in distribution and virulence phenotypes of *Heterodera glycines* in Missouri during 2005. Plant Dis. 91, 1473–1476. 10.1094/PDIS-91-11-1473 30780744

[B59] Navon O. Sul J. H. Han B. Conde L. Bracci P. M. Riby J.. (2013). Rare variant association testing under low-coverage sequencing. Genetics 194, 769–779. 10.1534/genetics.113.150169 23636738 PMC3697979

[B60] Nei M. Niimura Y. Nozawa M. (2008). The evolution of animal chemosensory receptor gene repertoires: roles of chance and necessity. Nat. Rev. Genet. 9, 951–963. 10.1038/nrg2480 19002141

[B61] Nelson B. D. Bradley C. A. (2003). Soybean cyst nematode (SCN). Fargo, ND: North Dakota State University Available online at: https://www.ndsu.edu/pubweb/~bernelso/soydiseases/cyst.shtml

[B62] Niblack T. L. Colgrove A. L. Colgrove K. Bond J. P. (2008). Shift in virulence of soybean cyst nematode is associated with use of resistance from PI 88788. Plant Health Prog. 10.1094/PHP-2008-0118-01-RS

[B63] Nikolic A. Kojic S. Knezevic S. Krivokapic Z. Ristanovic M. Radojkovic D. (2011). Structural and functional analysis of SMAD4 gene promoter in malignant pancreatic and colorectal tissues: detection of two novel polymorphic nucleotide repeats. Cancer Epidemiol. 35, 265–271. 10.1016/j.canep.2010.10.002 21036691

[B64] Novak S. J. (2007). The role of evolution in the invasion process. PNAS 104, 3671–3672. 10.1073/pnas.0700224104 17360409 PMC1820639

[B65] Pimentel D. Zuniga R. Morrison D. (2005). Update on the environmental and economic costs associated with alien-invasive species in the United States. Ecol. Econ. 52, 273–288. 10.1016/j.ecolecon.2004.10.002

[B66] Plantard O. Picard D. Valette S. Scurrah M. Grenier E. Mugniéry D. (2008). Origin and genetic diversity of Western European populations of the potato cyst nematode (*Globodera pallida*) inferred from mitochondrial sequences and microsatellite loci. Mol. Ecol. 17, 2208–2218. 10.1111/j.1365-294X.2008.03718.x 18410291

[B67] Pluess A. R. Frank A. Heiri C. Lalagüe H. Vendramin G. G. Oddou-Muratorio S. (2016). Genome–environment association study suggests local adaptation to climate at the regional scale in *Fagus sylvatica*. New Phytol. 210, 589–601. 10.1111/nph.13809 26777878

[B68] Plummer M. Best N. Cowles K. Vines K. (2006). CODA: convergence diagnosis and output analysis for MCMC. R News 6, 7–11. Available online at: http://oro.open.ac.uk/22547/

[B69] Poland J. A. Brown P. J. Sorrells M. E. Jannink J. L. (2012). Development of high-density genetic maps for barley and wheat using a novel two-enzyme genotyping-by-sequencing approach. PLoS ONE 7:e32253. 10.1371/journal.pone.0032253 22389690 PMC3289635

[B70] R Core Team (2017). R: A Language and Environment For Statistical Computing. Vienna, R Foundation for Statistical Computing Available online at: http://www.R-project.org/

[B71] Revell L. J. (2017). Phylogenetic Tools for Comparative Biology (and other things). Available online at: https://cran.r-project.org/web/packages/phytools/phytools.pdf

[B72] Riggs R. D. (2004). History and distribution, in Biology and Management of Soybean Cyst Nematode, 2nd Edn, eds Schmitt D. P. Wrather J. A. Riggs R. D. (Marceline, MO: Schmitt and Associates of Marceline), 9–40.

[B73] Rousset F. (1997). Genetic differentiation and estimation of gene flow from *F*-statistics under isolation by distance. Genetics 145, 1219–1228. 9093870 10.1093/genetics/145.4.1219 PMC1207888

[B74] Savage-Dunn C. Maduzia L. L. Zimmerman C. M. Roberts A. F. Cohen S. Tokarz R.. (2003). Genetic screen for small body size mutants in *C. elegans* reveals many TGFβ pathway components. Genesis 35, 239–247. 10.1002/gene.10184 12717735

[B75] Schlötterer C. Tobler R. Kofler R. Nolte V. (2014). Sequencing pools of individuals – Mining genome-wide polymorphism data without big funding. Nat. Rev. Genetics 15, 749–763. 10.1038/nrg3803 25246196

[B76] Shurtleff W. Aoyagi A. (2010). History of Soybeans and Soyfoods in Canada (1831–2010): Extensively Annotated Bibliography and Sourcebook. Lafayette, CA: Soyinfo Center.

[B77] Slack D. A. Riggs R. D. Hamblen M. L. (1972). The effect of temperature and moisture on the survival of *Heterodera glycines* in the absence of a host. J. Nematol. 4, 263–266. 19319277 PMC2619946

[B78] Statistics Canada (2017). Table 001–0017–Estimated Areas, Yield, Production, Average Farm Price and Total Farm Value of Principal Field Crops, in Imperial Units, Annual, CANSIM (Database). [Accessed: November 20, 2017]. Available online at: http://www5.statcan.gc.ca/cansim/a26?lang=eng&id=10017

[B79] Tardivel A. Sonah H. Belzile F. O'Donoughue L. S. (2014). Rapid identification of alleles at the soybean maturity gene E3 using genotyping by sequencing and a haplotype-based approach. Plant Genome 7, 1–9. 10.3835/plantgenome2013.10.0034

[B80] Tylka G. L. Marett C. C. (2017). Known distribution of the soybean cyst nematode, *Heterodera glycines*, in the United States and Canada: 1954 to 2017. Plant Health Prog. 18, 167–168. 10.1094/PHP-05-17-0031-BR

[B81] Van Tassell C. Smith T. P. Matukumalli L. K. Taylor J. F. Schnabel R. D. Taylor L.. (2008). SNP discovery and allele frequency estimation by deep sequencing of reduced representation libraries. Nat. Methods 5, 247–252. 10.1038/nmeth.1185 18297082

[B82] Watanabe N. Ishihara T. Ohshima Y. (2007). Mutants carrying two *sma* mutations are super small in the nematode *C. elegans*. Genes Cells 12, 603–609. 10.1111/j.1365-2443.2007.01077.x 17535251

[B83] Wood J. F. Ferkey D. M. (2016). GRK roles in C. *elegans*, in G Protein-Coupled Receptor Kinases, eds Gurevich V. V. Tesmer J. J. G. (New York, NY: Springer), 283–299.

[B84] Wright S. (1943). Isolation by distance. Genetics 28, 114–138. 17247074 10.1093/genetics/28.2.114 PMC1209196

[B85] Yu Q. (2011). Soybean cyst nematode (*Heterodera glycines* ichinohe), in Soybean Physiology and Biochemistry, ed El-Shemy H. A. (Rijeka: InTech Europe), 461–474. Available online at: http://cdn.intechopen.com/pdfs-wm/22782.pdf

[B86] Zheng J. Li Y. Chen S. (2006). Characterization of the virulence phenotypes of *Heterodera glycines* in Minnesota. J. Nematol. 38, 383–390. 19259544 PMC2586705

[B87] Zheng J. Z. Chen S. Y. (2011). Estimation of virulence type and level of soybean cyst nematode field populations in response to resistant cultivars. J. Entomol. Nematol. 3, 37–43. Available online at: http://www.academicjournals.org/journal/JEN/article-abstract/791181810204

